# Application of Mixture and Non-mixture Cure Models in Survival Analysis of Patients With COVID-19

**DOI:** 10.7759/cureus.58550

**Published:** 2024-04-18

**Authors:** Mohadese Kamalzade, Jamileh Abolghasemi, Masoud Salehi, Malihe Hasannezhad, Sadegh Kargarian-Marvasti

**Affiliations:** 1 Department of Biostatistics, School of Public Health, Iran University of Medical Sciences, Tehran, IRN; 2 Department of Infectious Diseases, Imam Khomeini Hospital Complex, Tehran University of Medical Sciences, Tehran, IRN; 3 Centers for Disease Control and Prevention, Health Center of Fereydunshahr, Isfahan University of Medical Sciences, Isfahan, IRN

**Keywords:** covid-19, akaike, log logistic, survival analysis, cure models

## Abstract

Background

Due to the emergence of new COVID-19 mutations and an increase in re-infection rates, it has become an important priority for the medical community to identify the factors affecting the short- and long-term survival of patients. This study aimed to determine the risk factors of short- and long-term survival in patients with COVID-19 based on mixture and non-mixture cure models.

Methodology

In this study, the data of 880 patients with COVID-19 confirmed with polymerase chain reaction in Fereydunshahr city (Isfahan, Iran) from February 20, 2020, to December 21, 2021, were gathered, and the vital status of these patients was followed for at least one year. Due to the high rate of censoring, mixture and non-mixture cure models were applied to estimate the survival. Akaike information criterion values were used to evaluate the fit of the models.

Results

In this study, the mean age of the patients was 48.9 ± 21.23 years, and the estimated survival rates on the first day, the fourth day, the first week, the first month, and at one year were 0.997, 0.982, 0.973, 0.936, and 0.928, respectively. Among the parametric models, the log-logistic mixed cure model with the logit link, which showed the lowest Akaike information criterion value, demonstrated the best fit. The variables of age and prescribed medication type were significant predictors of long-term survival, while occupation was influential in the short-term survival of patients.

Conclusions

According to the results of this study, it can be concluded that elderly patients should observe health protocols more strictly and consider receiving booster vaccine doses. The log-logistic cure model with a logit link can be used for survival analysis in COVID-19 patients, a fraction of whom have long-term survival.

## Introduction

In December 2019, several cases of respiratory illness caused by a viral infection were reported in Wuhan, China. In January 2020, the World Health Organization designated the virus responsible as the 2019 coronavirus and the illness it causes as COVID-19, declaring the outbreak a public health emergency [1]. These days, COVID-19 is considered the most dangerous member of the Coronaviridae family and a serious health threat globally [2]. One of the prominent characteristics of COVID-19 is its rapid spread. Up until the end of March 2024, there were more than 775 million contractions globally, nearly 7,043,660 of whom had succumbed to the disease [3]. In Iran, there were more than 7 million contractions and 146,811 deaths caused by COVID-19 [3]. The disease is usually asymptomatic, but some people may present with fever, dry cough, fatigue, nasal congestion, runny nose, sore throat, myalgia, and diarrhea. A significant proportion of infected patients develop pneumonia and severe acute respiratory failure, which usually require hospitalization in the intensive care unit (ICU) and intubation [4,5]. Many studies have shown that comorbidities such as diabetes, cardiac disease, hypertension, pulmonary problems, cerebrovascular disorders, advanced age, male gender, smoking, and obesity can be predictors of mortality in COVID-19 patients. According to previous studies, the most common comorbidities associated with COVID-19 include hypertension (17%), diabetes mellitus (8%), and cardiovascular diseases (5%) [4].

Another study noted that elderly male patients with underlying diseases were more likely to develop severe disease progressing to pneumonia [6]. Semi-parametric and parametric models can be used to predict the factors affecting survival in COVID-19 patients. A condition required to be able to use these models is that the loss of all subjects under study occurs gradually and over a long period; however, this condition is not always met, implying that a certain proportion of patients may not experience the desired outcome during the follow-up period, so conventional parametric and semi-parametric models cannot be used. For example, not all people infected with human immunodeficiency virus will develop acquired immunodeficiency syndrome. From a scientific point of view, it is important to distinguish between cured people and censored cases vulnerable to developing the desired outcome. In fact, a high rate of censoring in survival data causes bias in predicting maximum likelihood estimates when using various survival standard models. Cure models can resolve these limitations and be applicable to cases where classical statistical models are not appropriate [7].

One of the important goals of cure models is to estimate the ratio of cured (safe) people, obtain the survival function for at-risk, vulnerable social groups, and identify the factors influencing these parameters, where these models can actually offer a better interpretation. The presence of cured individuals in a data sample is usually represented by a Kaplan-Meier curve, demonstrating a long surface leveled with dense censoring (long-term censoring) at its far-right [8].

It is important to detect the risk factors of mortality early to better predict a disease’s clinical and epidemiological characteristics and to enable receiving appropriate supportive care and timely special care if necessary [9]. Therefore, this study aimed to identify factors affecting the time of death in patients with COVID-19 using mixture and non-mixture cure models.

## Materials and methods

The data gathered in this research belonged to 880 patients with a confirmed diagnosis of COVID-19 based on polymerase chain reaction (PCR) testing registered in the health center of Fereydonshahr City, Isfahan, from February 20, 2020, to December 21, 2021, who were followed up for at least one year. In this survival analysis study, the outcome variable was the duration (days) from the diagnosis of COVID-19 by PCR until the time of the patient’s death. Censored cases included those who survived until the end of the study. The data needed, including age, gender, inpatient or outpatient status, administered drugs, the time of the onset of symptoms, time of sampling, time of death, the interval between the onset of symptoms and visiting a physician’s office or death, occupation, education level, ethnicity, marital status, economic status, history of underlying diseases (hypertension, diabetes, cardiac disease, pulmonary disorders, neuronal conditions, renal dysfunction, and liver disease), access to health services, place of residence, the route of disease contraction, and the survival status, were gathered by reviewing patients’ profiles and, if required, via face-to-face or phone interviews. In this study, the criterion used to categorize disease severity was the type of the drugs administered by a specialist (i.e., no medication: asymptomatic or very mild disease; symptom-directed medications: moderate severity without the need for hospitalization; specialized antiviral medications: severe disease requiring prompt hospitalization).

Akaike information criteria (AIC) was used to compare the fit of the models. Suppose that we have a statistical model of some data. Let k be the number of estimated parameters in the model. Let $\hat{L}$ be the maximized value of the likelihood function for the model. Then the AIC value of the model is $AIC= 2K-2\hat{L}$ [10]. The smaller this criterion is, the better fit the proposed model possesses.

Cure models

Cure models used for survival analysis are divided into two categories, namely, mixture and non-mixture models. Mixture cure models consist of the cured (i.e., patients who are immune to the desired occurrence) and non-cured (i.e., patients with no immunity to the desired occurrence and threatened by its risk until the end of the study period) groups. The survival function is defined as follows [11]:

$S(t)=&pi;+(1-&pi;) S^* (t), S^* (&infin;)=0$ (1)

Indeed, the cure fraction, derived from a binary model such as logistic regression, indicates the probability of an individual being assigned to either the cured or non-cured group. For instance, when employing a logit link function, the probability of an individual belonging to the cure group is expressed as follows:

$ln⁡(&pi;/(1-&pi;))= &alpha;_0+&alpha;_1 X_1+⋯+&alpha;_c X_c$ (2)

$\hat{\pi }= \frac{1}{1+exp-(\alpha _{0}+\alpha _{1}x _{1}+...+\alpha _{k}x _{k})}$

In this model, π represents the proportion of cured (or immune) subjects, which can be modeled using logistic regression, the probit function, the log-log complement link function, and linear regression. The function S*(t) also indicates the survival function of the non-immunized group. xs represent the significant variables in the logistic model, while αs denote the regression coefficients of this model. Additionally, c represents the number of variables that have become statistically significant in the final logistic model.

When considering a specific distribution such as exponential, Weibull, or log-logistic for the time to event, the resulting function S*(t) is presented in Table 1 [12].

**Table 1:** Survival functions and regression coefficients of exponential, Weibull, log-logistic, and log-normal models.

Distribution	S*(t)	Estimation of coefficient
Exponential	$exp({-\lambda t}$)	$\lambda =exp(\beta _{0} +\beta _{1} X_{1} + ... + \beta _{K} X_{K})$
Weibull	$exp({-\lambda t^{p}}$)	$\lambda =exp(\beta _{0} +\beta _{1} X_{1} + ... + \beta _{K} X_{K})$
Log-logistic	$\frac{1}{1+\lambda t^{p}}$	$\lambda = exp (\beta _{0}+ \beta _{1} X_{1} + ... + \beta _{K} X_{K})^{-p}$
Log-normal	$1-\Phi [\frac{lnX-\mu }{\sigma }]$	$ln(t) = \beta _{0}+ \beta _{1} X_{1} + ... + \beta _{K} X_{K}$

Further, t represents the time to the event. λ and p are distribution parameters. λ undergoes reparameterization and determines the values of regression coefficients (βs). In the log-normal distribution, μ and σ are the mean and standard deviation, respectively, and Φ is the cumulative distribution function (CDF) of the standard normal distribution.

Non-mixture cure models are defined for populations with an incomplete distribution function for the survival of all individuals in the population (i.e., a value of <1 for the cumulative distribution function). The survival function for these models is defined as noted below [13]:

$S(t)=\pi ^{1-S^{*}(t)}$ (4)

For survival analysis, we applied mixture and non-mixture exponential cure models, as well as Weibull, log-logistic, and log-normal models. Moreover, the significance level was considered at 0.05 (the significance level was 0.2 for single regression), and the logit function was regarded as the link function. For the fitting of the multiple cure model, we adjusted the scale parameter (λ) and various combinations of variables in the cure fraction and shape (p). This adjustment resulted in improved fitting for each of the presented distributions, i.e., Weibull, log-normal, log-logistic, and normal. The selection was based on achieving a lower AIC value, indicating a favorable model fit. The modeling process was conducted iteratively, employing both forward and backward selection procedures. This approach allows for the identification of the most significant variables and their optimal combination for the scale, cure fraction, and shape parameters. Data analysis was conducted in R software (version 4.3.2).

## Results

In this study, of 880 patients with COVID-19, 445 (50.57%) were male, 668 (75.91%) were married, 121 (13.75%) were single, and 568 (64.55%) were literate. The overall mean age was 48.9 ± 19.60 years, and the mean ages of outpatients, inpatients, and deceased patients were 44.85 ± 19.56, 65.64 ± 21.23, and 75.08 ± 15.06 years, respectively. Based on disease severity and pulmonary involvement in lung scans, the patients were divided into two groups, namely, inpatients and outpatients. According to the results, 707 (80.34%) patients were outpatients, and 173 (19.66%) required specialized antiviral medications. Of the hospitalized patients who received specialized antiviral drugs, 104 (60.12%) had at least one comorbidity, whose mean age was 63.72 ± 16.13 years. Table 2 summarizes the data of the patients investigated based on their final outcomes (i.e., death and censored). The estimated survival rates on the first day, the fourth day, the first week, the first month, and at one year were 0.997, 0.982, 0.973, 0.936, and 0.928, respectively.

**Table 2:** Determining and comparing the descriptive indices of demographic and clinical variables separately for death and alive.

Variables		Alive	Death	Statistic X^2^	P-value
		Frequency (%)	Frequency (%)		
Gender	Male	415 (50.7)	30 (48.4)	0.127	0.722
	Female	403 (49.3)	32 (51.6)		
Marital status	Married	624 (76.3)	44 (70.97)	26.173	<0.001
	Single	120 (14.7)	1 (1.61)		
	Widow	74 (9.0)	17 (27.42)		
Hospitalization status	Outpatients	692 (84.6)	15 (24.19)	133.131	<0.001
	Hospitalized	126 (15.4)	47 (75.81)		
Ethnicity	Lur	227 (27.8)	19 (30.60)	1.496	0.683
	Persian	144 (17.6)	11 (17.70)		
	Turkish	113 (13.8)	11 (17.70)		
	Georgian	334 (40.8)	21 (33.90)		
Education	Illiterate	260 (31.78)	52 (83.87)	68.323	<0.001
	Literate	558 (68.22)	10 (16.13)		
Job environment	Home	419 (51.22)	53 (85.49)	30.458	<0.001
	Indoor	250 (30.56)	1 (1.61)		
	Outdoor	149 (18.22)	8 (12.90)		
Treatment type	No treatment	659 (80.60)	8 (12.90)	148.442	<0.001
	Symptomatic medications	33 (4.00)	7 (11.29)		
	Specialized antiviral drugs	126 (15.40)	47 (75.81)		
Economic status	Weak	151 (18.50)	23 (37.10)	20.886	<0.001
	Moderate	139 (39.00)	29 (46.77)		
	Good	348 (42.50)	10 (16.13)		
Underlying disease	Yes	236 (28.90)	42 (67.74)	40.335	0.001
	No	582 (71.10)	20 (32.26)		
Ways of disease transmission	The presence of a positive case in the family	268 (32.76)	12 (19.35)	14.49	0.002
	Non-compliance with protocols	186 (22.74)	20 (32.26)		
	Occupational contact	196 (23.96)	8 (12.90)		
	Travel and participation in public ceremonies	168 (20.54)	22 (35.48)		
Residency status	Rural	284 (34.72)	29 (46.77)	3.655	0.056
	Urban	534 (65.28)	33 (53.23)		
Service access status	Within one hour	740 (90.46)	50 (80.65)	6.052	0.014
	Over one hour	78 (9.54)	12 (19.35)		
		Mean ± SD	Mean ± SD	t	P-value
Age		46.2 ± 20.31	75.1 ± 15.06	10.684	<0.001

**Figure 1:**
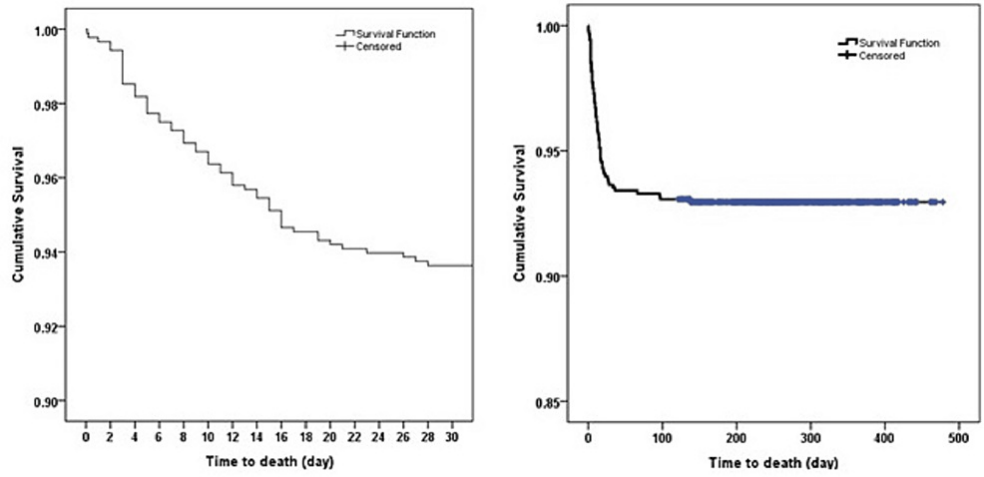
Kaplan-Meier survival curve for COVID-19 patients 30 and 500 days after diagnosis.

Figure 1 shows a Kaplan-Meier curve with a censoring rate beyond 0.9 for the dataset of this study, necessitating the use of cure models for survival analysis.

According to the chi-square test, the mortality rate in hospitalized patients was three times higher compared to outpatients (p < 0.001). Moreover, there was a significant difference in the mortality rate regarding the type of treatment received (p < 0.001) (i.e., no treatment, symptom-directed treatment, and specialized antiviral treatment). In this regard, of 173 patients who received specialized medications, 47 (75.8%) died, which was significantly higher compared to the respective ratios in those who received no treatment (12.9%) or symptom-directed treatment (11.3%). In this study, the mortality rate was not significantly associated with gender and ethnicity, but it showed a significant correlation with marital status and occupation (p < 0.001). Moreover, the mortality rate in patients who had at least one comorbidity was more than twice that of patients with no underlying condition, and this difference was also statistically significant (p < 0.001). The results of the independent t-test are shown in Table 2, indicating a significantly higher mean age in patients who died than in those who survived (p < 0.001).

In survival analysis using a single regression variable for four mixture and non-mixture cure models, the variables of age, disease status, type of treatment, economic status, job, education level, route of disease transmission, the presence of comorbidities, service access status, and residency status achieved a significance level of 0.2.

Following single regression, multiple regression was performed for mixture and non-mixture cure models to assess their fitness by applying forward and backward methods. The lowest AIC was related to the mixture log-logistic cure model (741.24), indicating its better fit compared to other models. Akaike values resulting from multiple regression are shown in Table 3. Table 4 presents the results of multiple regression for the log-logistic mixture cure model, reflecting the significant effects of age and the type of treatment on the long-term survival of patients. Based on these variables, the patients were divided into the following two groups: cured and non-cured. By reducing one year of age, the chance of being in the cured group increases by 1.06 times, and by decreasing by 10 years (e.g., a 30-year-old person compared to a 40-year-old), the chance increases by 1.93 times (i.e., nearly twice). Further, the odds of being in the cured group was 8.85 times of patients who did not use drugs than in patients who used specialized drugs, but in the uncured group, the ratio of inpatients to outpatients at risk of death was approximately 2.5 times.

**Table 3:** Akaike values for multiple regression of the mixture and non-mixture cure model.

Distribution	Cure model	
	Mixture	Non-mixture
Exponential	754.62	759.95
Weibull	748.54	750.52
Log-logistic	741.24*	747.44
Log-Normal	754.52	751.46

**Table 4:** Results of the fitted multiple regression by the log-logistic mixture cure model.

	Variable		β	SE	P-value	95% CI
Cure fraction	Age		-0.066	0.013	0.001	-0.093, -0.039
	Treatment type	No treatment (base)	-	-	-	-
		Symptomatic medications	-0.828	0.640	0.196	-2.082, 0.427
		Specialized antiviral drugs	-2.150	0.449	<0.001	-3.037, -1.275
	Constant		7.769	0.892	<0.001	0.519, 6.020
Scale	Hospitalization status	Outpatients (base)	-	-	-	-
		Hospitalized	0.688	0.374	0.066	-0.045, 1.421
	Constant		-2.811	0.336	<0.001	-3.470, -2.151
Shape	Constant		0.406	0.118	<0.001	0.173, 0.639

## Discussion

In this study, the survival of COVID-19 patients was significantly associated with age as a discriminator between immune and vulnerable patients (p = 0.001). In this regard, an increase in age correlated with a reduction in the cure rate, which was consistent with previous reports affirming a significant association between age and the mortality rate in elderly patients with COVID-19 [14]. Several factors can contribute to the severity of COVID-19 and its high mortality in older patients. One of these factors is the susceptibility of elderly people to infections, which can be explained by a phenomenon known as immunosenescence. With increasing age, T and B lymphocytes decline in quantity, devitalizing the body’s natural immunity over time. An inefficient immune system can lead to deregulated immune responses, and the overactivation of immune cells ensues with the excessive production of cytokines, resulting in a cytokine storm and inflammation in the body of patients. Moreover, dysfunctional innate and acquired immunity systems lead to chronic clinical systemic inflammation (i.e., inflammaging), characterized by elevated proinflammatory markers such as interleukin 6 and C-reactive protein and elevated susceptibility to infections. Of note, inflammation is a key pathogenic mechanism contributing to the poor outcomes of elderly patients with COVID-19 [15].

As the SARS-CoV-2 is transmitted mainly through the respiratory tract, patients’ lungs are severely damaged by COVID-19, which is partly attributed to the high expression of angiotensin-converting enzyme 2 (ACE2) by pulmonary alveolar type 2 (AT 2) cells, accounting for one of the main reasons behind the tendency of the SARS-CoV-2 to spread in the lung. Studies have shown that the main cellular targets of this virus include airway epithelial cells, vascular endothelial cells, and pulmonary macrophages. The binding of the SARS-CoV-2 to the ACE2 receptor leads to the overactivation of the immune system in some patients, promoting an exaggerated immune response in the lungs, followed by a cytokine storm, and, subsequently, pulmonary edema, disruption of air exchange in the lung, and acute respiratory distress syndrome (ARDS) [16]. The physiological and pathophysiological functions of the lungs during infections change with aging [17], which is associated with ineffective clearance of airways, decreased lung reserve, the compromised function of protective barriers, and, finally, a reduction in the responsiveness and tolerance of elderly patients [18]. Therefore, older patients are likely to show lower tolerance to COVID-19 and may succumb to the disease sooner than younger individuals.

In addition, ACE2 receptors are expressed on cardiac myocytes, so the interaction between the SARS-CoV-2 virus and these receptors can lead to direct myocyte toxicity [19]. Respiratory viral infections can trigger pathways that facilitate vascular complications, including direct contamination of myocardial cells mediated via ACE, which is believed to partly play a role in SARS-COV2-induced cardiac damage. Moreover, the activation of hemostatic and inflammatory pathways can pave the path for the development of new vascular complications or the exacerbation of preexisting cardiovascular diseases [20].

The cytokine storm during the COVID-19 infection triggers the activation of Th1 and Th17 lymphocytes, leading to systemic inflammation and the infiltration of the lungs and heart with lymphocytes and proinflammatory monocytes, the outcomes of which include ARDS, ARDS-induced hypoxia, acute cardiac injury (ACI), or the progression of ACI to heart failure [16]. Various studies have confirmed that SARS-CoV-2-infected elderly patients are more vulnerable to cardiac complications and are more likely to experience ARDS, cardiac injury, hospitalization, and in-hospital mortality [17,18].

Another concerning issue in elderly patients is the cardiotoxicity caused by antiviral treatments. The prescription of ACE inhibitors or angiotensin receptor blockers should be with caution in patients with cardiovascular disease as these medications can upregulate ACE2 and potentially aggravate the patient’s health outcome [20,21]. It is worth mentioning that older people are also vulnerable to drug side effects, partly because of either a reduction in the functionality of their organs or receiving several medications due to other comorbidities [22]. In a retrospective cohort study, it was stated that the number of drugs consumed before admission was higher in patients who died during hospitalization than in patients who were discharged [23].

Another contributor to the higher mortality and morbidity rates in older patients can be the higher average number of comorbidities, showing a positive persistent correlation with age [15,24]. Generally, all individuals are vulnerable to contracting COVID-19; however, this risk is exaggerated in elderly people, particularly those with comorbidities, who are more likely to develop severe complications or die due to this disease [18,25,26]. In this study, the univariate cure model revealed that the presence of at least one comorbidity could significantly boost the risk of death in COVID-19 patients.

In this study, another risk factor for COVID-19-related mortality was identified to be the need for the administration of specialized antiviral medications (p < 0.001). Regarding the association between the type of treatment and mortality in COVID-19 patients, one can mention the role of disease severity, where patients with more severe conditions are more likely to benefit from specialized medications but also have a higher chance for hospitalization and in-hospital mortality. In the present study, 173 hospitalized patients showed a more severe disease and a higher need for receiving specialized antiviral medications.

COVID-19 patients may develop liver dysfunction due to the side effects of the drugs used to treat the infection. In a systematic review by Mohammadi et al., COVID-19 patients with abnormal liver function tests received lopinavir/ritonavir at a 25% higher rate than patients with normal liver function tests. Liver impairment can lead to dire consequences and extend the period of hospitalization due to immune system dysfunction, especially in elderly patients with numerous comorbidities [27].

In the present study, the patient’s status (i.e., inpatient or outpatient) was identified as an important predictor of COVID-19-related mortality (p = 0.053). In this regard, hospitalized COVID-19 patients had an almost 2.5-fold higher chance of dying compared to outpatients, which can be explained by the fact that inpatients generally suffer from a more severe disease and, therefore, are more likely to die compared to outpatients. In addition, we observed that the mean age of hospitalized patients was over 65 years, which can be an explanation for their higher mortality. A study by Liu et al. showed that elderly COVID-19 patients had a higher ratio of multi-lobe involvement, more need for mechanical ventilation, and a higher incidence of ARDS and mortality compared to young and middle-aged people [18]. Moreover, a population-based study in Brazil found an association between age and the mortality rate in hospitalized COVID-19 patients [28]. An international multicenter study in Europe showed that hospitalized COVID-19 patients with respiratory symptoms had significantly shorter survival compared to other patients [29].

Study limitations

The presence of missing data and lack of access to samples were among the study limitations. The researchers tried to contact the patients through phone calls and text messages to reduce the limitations.

## Conclusions

According to the findings of this study, elderly patients are suggested to follow health protocols more strictly and consider booster doses of COVID-19 vaccines. Further, healthcare providers should pay close attention to drug side effects in COVID-19 patients, particularly in elderly patients, due to a higher likelihood of polypharmacy. Our findings showed that the patient’s hospitalization status (i.e., inpatient or outpatient) was a significant contributor to COVID-19-related mortality; hence, early visits to a doctor could substantially prevent or reduce the adverse consequences of this infection, including mortality. Regarding the efficiency of the mixture log-logistic cure model in predicting the factors affecting the survival of COVID-19 patients, considering that this model showed a sharp increase and then a declining trend in the desired outcome, it can be concluded that in non-cured patients, the mortality would be at its zenith in early days after the infection, followed by a reduction in the number of deaths afterward.
